# Immunogenomic profiling reveals targets for gene therapy in pediatric brain tumors

**DOI:** 10.1093/noajnl/vdag193

**Published:** 2026-07-27

**Authors:** Giulia Rovesti, Alejandro Fernandez Woodbridge, Haidong Yao, Alessia Pancaldi, Chiara Chiavelli, Francesca Gatto, Ulrika Sandvick, Matti Sällberg, Jonas Fuxe, Massimo Dominici, Hans Grönlund, Ola B Nilsson, Daniela Nascimento Silva, Anna Pasetto

**Affiliations:** Department of Oncology and Haematology, Azienda Ospedaliero-Universitaria di Modena, Modena, Italy; NEOGAP Therapeutics AB, Stockholm, Sweden (A.F.W., H.G., O.B.N.); NEOGAP Therapeutics AB, Stockholm, Sweden (A.F.W., H.G., O.B.N.); Department of Laboratory Medicine, Karolinska Institutet, Stockholm, Sweden; Department of Medical and Surgical Sciences of Children and Adults, University of Modena and Reggio Emilia, Modena, Italy; Department of Laboratory Medicine, Karolinska Institutet, Stockholm, Sweden; Department of Clinical Neuroscience, BioClinicum, Karolinska Instituet, Solna, Sweden; Department of Neurosurgery, Karolinska University Hospital, Solna, Sweden; Department of Laboratory Medicine, Karolinska Institutet, Stockholm, Sweden; Department of Laboratory Medicine, Karolinska Institutet, Stockholm, Sweden; Department of Oncology and Haematology, Azienda Ospedaliero-Universitaria di Modena, Modena, Italy; Department of Medical and Surgical Sciences of Children and Adults, University of Modena and Reggio Emilia, Modena, Italy; Therapeutic Immune Design, Centre for Molecular Medicine, Department of Clinical Neuroscience, Karolinska Institutet, Stockholm, Sweden; NEOGAP Therapeutics AB, Stockholm, Sweden (A.F.W., H.G., O.B.N.); Department of Laboratory Medicine, Karolinska Institutet, Stockholm, Sweden; Section for Cell Therapy, Radiumhospitalet, Oslo University Hospital, Oslo, Norway; Karolinska ATMP Center, Karolinska Institutet, Stockholm, Sweden; Department of Laboratory Medicine, Karolinska Institutet, Stockholm, Sweden; Pediatric Oncology and Hematology Unit, Department of Medical and Surgical Sciences of Children and Adults, Azienda Ospedaliero-Universitaria di Modena, Modena, Italy; Clinical and Experimental Medicine PhD Program, University of Modena and Reggio Emilia, Modena, Italy; Section for Cell Therapy, Radiumhospitalet, Oslo University Hospital, Oslo, Norway; Department of Oncology, Institute of Clinical Medicine, University of Oslo, Oslo, Norway; Karolinska ATMP Center, Karolinska Institutet, Stockholm, Sweden

**Keywords:** CAR-T cells, neoantigen discovery, pDMG, TCR-T cells, tumor microenvironment

## Abstract

**Background:**

Pediatric brain tumors, particularly pontine diffuse midline glioma (pDMG), remains lethal with limited therapeutic options. Improved stereotactic biopsy techniques and advances in bioinformatics are progressively enabling deeper exploration of immunological vulnerabilities empowering novel strategies, including adoptive cell and gene therapies (ACGTs). We aimed to integrate genomic, transcriptomic, and immunological analyses to identify actionable pathways, surface antigens, and neoantigens that could inform next-generation immunotherapies, including Chimetic Antigen Receptor (CAR)-T cells and T cell Receptor (TCR)-T cell strategies.

**Methods:**

Primary pDMG samples (*n* = 6) underwent whole-genome and RNA sequencing. Transcriptional drug response profiling was used to define targetable transcriptional dependencies. Surface antigen expression and immune cell composition were assessed to evaluate suitability for ACGTs. Neoantigen prediction employed PIOR, integrating somatic variant calling, Human Laukocyte Antigen (HLA) binding, and expression data. Prioritized neoantigens were synthesized and used to stimulate healthy donor T cells. Activated CD137^+^ T cells were sorted for bulk TCR sequencing to identify clonally expanded TCRs.

**Results:**

Transcriptional drug response profiling revealed heterogeneous but actionable pathway dependencies. B4GALNT1 expression varied across tumors, identifying a subset with GD2 levels compatible with CAR-T targeting. Tumor microenvironment profiling showed enrichment of dendritic cells and M2 macrophages, with scarce CD8^+^ T cells and NK cells. Across samples, 31 somatic variants were identified including alterations in *ACVR1*, *H3K27M*, and *TP53*. Several predicted neoantigens induced T cell activation and clonal expansion.

**Conclusion:**

This integrated profiling approach identifies targetable pathways, surface antigens, and neoantigens in pDMG, supporting the development of CAR-T and TCR-T therapies. These insights also suggest potential applicability to other cancers harboring shared mutations.

Key Points

- pDMG shows targetable pathway dependencies and heterogeneous antigen expression.
- Neoantigen stimulation revealed clonal expansion suggestive of T cell reactivity.
- Findings support development of ACT strategies tailored to pDMG vulnerabilities.

Importance of the Study

Pontine diffuse midline glioma remains a uniformly fatal pediatric brain tumor with no effective targeted therapies and minimal benefit from conventional treatment modalities. In this study, we integrated whole-genome and transcriptomic profiling with immunogenomic analyses to systematically identify molecular vulnerabilities, surface antigen heterogeneity, and neoantigen candidates in a unique cohort of primary pontine diffuse midline glioma (pDMG) samples. Our findings highlight the complex mutational landscape, limited immune infiltration, and suppressive tumor microenvironment that constrain current therapeutic approaches. By uncovering targetable pathway dependencies, variable GD2 expression, and neoantigens capable of eliciting T-cell activation and clonal expansion, we provide a framework for developing rational, personalized immunotherapeutic strategies. This multi-layered analysis underscores the need for combinatorial approaches that integrate cellular therapies, pathway-directed interventions, and microenvironment-modulating strategies to overcome the biological barriers inherent to pDMG.

Diffuse intrinsic pontine glioma, classified in the 2021 WHO CNS5 update as “pontine diffuse midline glioma, H3 K27M-mutant, grade 4” (pDMG), is among the most lethal pediatric brain tumors, typically presenting at 6-8 years of age and carrying a median survival of approximately 11 months.^1-4^ Its infiltrative brainstem location precludes surgical resection and limits the effects of radiotherapy and chemotherapy, and despite 4 decades of clinical trials, survival has not substantially improved.^5^^,^^6^ Radiotherapy remains the only established standard of care, providing transient clinical or radiographic responses in many patients; re-irradiation is often considered at progression, whereas multiple chemotherapeutic strategies have failed to achieve durable disease control.^2^^,^^6^^,^^7^ Although adjuvant chemotherapy is widely used, no optimal regimen has been established; the 2025 SIOPE HGG Working Group recommended chemoradiation with temozolomide followed by 6-12 cycles of adjuvant temozolomide, reflecting modest event-free survival (EFS) benefit but no overall survival improvement in HIT-HGG-2007.^8^ Progress has historically been constrained by limited tumor tissue, as biopsies were often avoided because of perceived risk; however, stereotactic biopsy is now considered feasible and safe in experienced centers, enabling molecular profiling of diagnostic and trial-derived material.^9-13^ Molecular studies have identified recurrent, potentially targetable alterations, including H3K27M mutations, ACVR1 mutations, PI3K/AKT/mTOR pathway dysregulation, and PDGFRA amplification or mutation, which have motivated trials of epigenetic, bone morphogenetic protein (BMP)-signaling, PI3K/mTOR, PDGFRA-directed, and other biologically informed therapies.^14-24^ Additional strategies under investigation include blood-brain barrier-disrupting or bypassing drug-delivery approaches, immunotherapies, and combinatorial treatment concepts designed to overcome tumor heterogeneity and treatment resistance.^25-29^ A complementary strategy for identifying therapeutic T cell receptors (TCRs) has been developed by Steven Rosenberg’s group at the NIH, in which activation-induced CD137/4-1BB expression is used to enrich tumor- and neoantigen-reactive T cells from tumor-infiltrating lymphocytes^30^^,^^31^ and peripheral blood^32^ after antigen exposure. This approach has enabled isolation and cloning of mutation-reactive TCRs, including TCRs targeting patient-specific neoantigens and shared oncogenic mutations, which can then be introduced into autologous peripheral blood lymphocytes to generate TCR-engineered T cells for adoptive cell therapy.^31^^,^^33^^,^^34^ Importantly, this concept has now advanced clinically, with personalized neoantigen-reactive TCR-transduced T cells showing feasibility and early antitumor activity in metastatic colorectal cancer, supporting the translational relevance of identifying and cloning endogenous tumor-reactive TCRs.^35^ Therefore, the rationale for this study is to characterize clinically relevant molecular alterations in a national Swedish pDMG cohort to identify actionable vulnerabilities and support future precision-medicine strategies.

## Methods

### Patient Samples

Sequencing data of pDMG samples were obtained from the Swedish Childhood Tumor Bank (Barntumorbanken) and the utilization of the sequencing data for research purposes was also approved by the Swedish Ethical Review Authority (2022-04022-02). The cohort comprised stereotactic diagnostic tumor biopsies collected fresh (not post-mortem) and matched blood samples. Six patients with pDMG were included in this study, each providing tumor tissue and matching peripheral blood lymphocytes (PBL) as germline controls.

### Nucleic Acid Extraction and Sequencing

Nucleic acids were extracted from fresh biopsies and sequenced at SciLifeLab-Solna following Qiagen (QIAseq Human Exome Kit (24) Cat. No./ID: 333937) and Illumina protocols. Whole-genome sequencing (WGS) libraries were generated using the TruSeq DNA PCR-Free kit and sequenced on the Illumina NovaSeq 6000 to a target depth of 90 × (tumor) and 30 × (nonmalignant). RNA-sequencing (RNA-seq) libraries were prepared with the TruSeq Stranded Total RNA kit (Ribo-Zero Gold) and sequenced to ∼100 million paired-end reads per sample.

### Identification of Somatic Variants and Neoantigen Prediction

WGS and RNA-seq data from tumor and matched blood samples were processed using the PIOR pipeline.^36^^,^^37^ Following FastQC quality assessment, reads were aligned to the GRCh38 reference genome using Burrows–Wheeler Aligner–Maximal Exact Matches (BWA-MEM) and processed with SAMtools for sorting, duplicate marking, and indexing. High-confidence somatic variants were identified, annotated using Variant Effect Predictor (VEP) to predict functional consequences, ranked, and visually inspected. Variant-derived 25 mer peptide sequences harboring identified with PIOR were subsequently analyzed using the Ardigen platform, including the ArdImmune Vax algorithm, to predict Humand Leukocyte Antigen (HLA) presentation and minimal epitope candidates. Minimal epitopes were defined through iterative peptide truncation and recalculation of predicted binding affinity. High-affinity epitopes were selected for downstream functional validation and synthesized (GenScript). Corresponding sequences are listed in Supplementary Table S1.

### Transcriptomic Data Acquisition, Processing, and Drug Response Prediction

To predict patient-specific drug responses, bulk RNA-seq count data were compiled from publicly available and in-house cohorts, including the Genotype-Tissue Expression (GTEx) project, The Cancer Genome Atlas liver hepatocellular carcinoma cohort (TCGA-LIHC), and the Open Pediatric Brain Tumor Atlas (OpenPBTA).^38^ Additional datasets from the Cancer Dependency Map (DepMap) and Drug Sensitivity Screening (DSS) resources were used as pharmacogenomic training data for drug-response modeling.^39^

**Figure 1.**
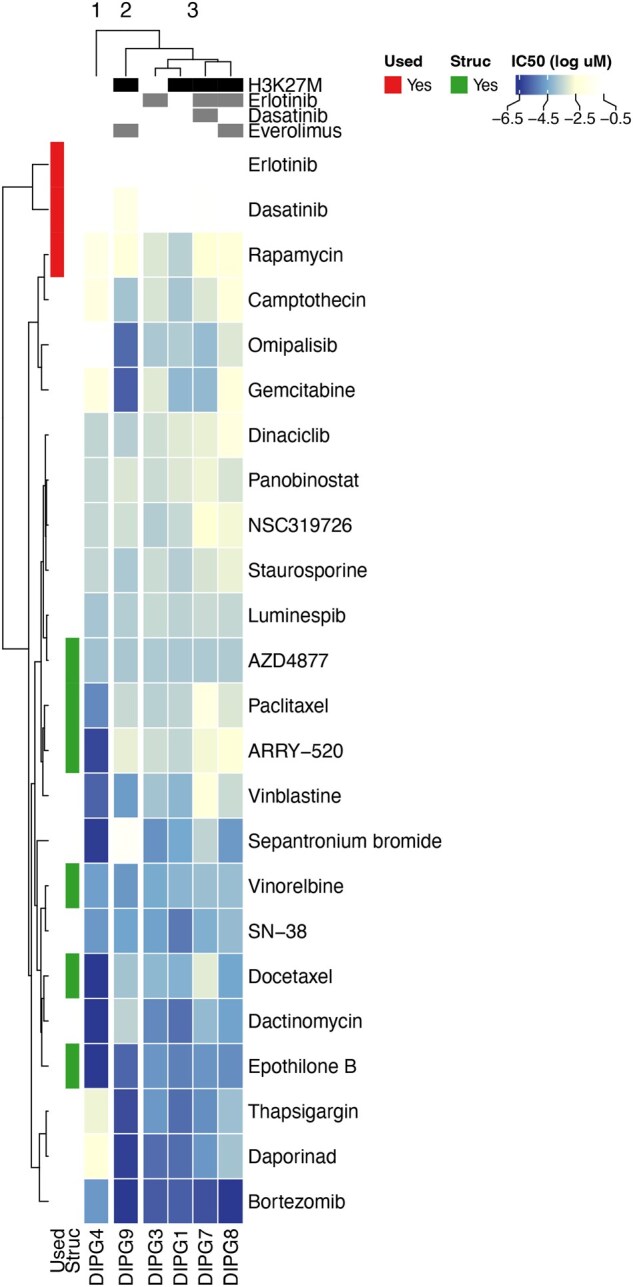
Integrative pharmacotranscriptomic profiling of pDMG samples. Heatmap depicting predicted drug sensitivity IC_50 scores derived from tumor RNA-seq profiles via oncoPredict. Sensitivity is represented on a color gradient where low (blue) values indicate high predicted response and high (white) values indicate resistance. Row annotations highlight compounds targeting microtubule stability or spindle formation (green) and drugs utilized in clinical treatment (red); grey markers specifically denote the administration of Erlotinib, Dasatinib, or Everolimus (proxied by Rapamycin sensitivity). The analysis reveals inter-patient heterogeneity, notably showing that patients with the highest predicted sensitivity to Rapamycin were not treated with Everolimus, while treated patients exhibited diminished predicted responses. Furthermore, the screen identifies a cluster of antimitotic agents as potential synthetic lethality candidates, likely exploiting chromatin compaction instabilities induced by the H3K27M mutation. These findings recover established treatments like Panobinostat while identifying novel therapeutic avenues for H3K27M-mutant pDMG.

For all datasets, raw or processed transcriptomic count matrices were harmonized prior to downstream analysis. Ensembl gene identifiers were converted to HUGO Gene Nomenclature Committee (HGNC) protein-coding symbols using the biomaRt R package.^40^ To minimize redundancy introduced by transcript or gene identifier mapping, duplicate gene entries were collapsed by retaining the transcript or gene with the highest expression. Specifically, transcripts of the same gene across approximately 60,000 genes were normalized to reads per kilobase per million mapped reads (RPKM) and log2-transformed. The final merged expression matrices contained genes consistently annotated and represented across all study cohorts and pharmacogenomic training datasets. In vivo pharmacogenomic training data were sourced from the Cancer Dependency Map Drug Repurposing dataset (Broad Institute, n.d.). This resource contains paired bulk cell-line RNA-seq profiles and multi-dose drug-response data, including area under the curve values and compound-sensitivity scores. To construct a robust training set, compounds with limited response variability were removed, including those with close-to-zero response values across most cell lines and those with insufficient representation across the dataset. This standardized training pipeline was used for ridge-regression-based drug-sensitivity prediction. Drug-response prediction for clinical and normal-tissue samples was performed using the oncoPredict R package.^41^ The algorithm extends the pRRophetic framework^42^ by training ridge-regression models that relate baseline gene expression in pharmacogenomic reference cell lines to measured drug response. These trained models were then applied to normalized RNA-seq expression profiles from clinical cohorts, GTEx normal tissues, and pediatric brain tumor datasets to infer relative drug sensitivity. Predicted drug-response values were generated for each sample and compound, producing sample-by-compound matrices for downstream analysis. For analysis based on the DSS pharmacogenomic resource, drug-sensitivity prediction was performed using a comparable oncoPredict workflow. Drug-response phenotypes and matched transcriptomic profiles were obtained from DSS, and models were trained after filtering for compounds with sufficient variability and sample coverage. To align the prediction space across datasets, common genes between the pharmacogenomic training set and the target expression matrices were retained. For large-scale prediction, compounds were processed in batches to reduce computational demand, with approximately 1000 compounds included per input batch where applicable. Differential predicted drug sensitivity was assessed across predefined molecular or clinical groups using the compareDrugs function in oncoPredict, with statistical significance evaluated using Wilcoxon rank-sum testing and correction for multiple comparisons. Predicted sensitivity scores, cohort-level patterns, and ranked drug candidates were visualized using R-based workflows, including tidyverse-compatible data wrangling and plotting. The overall transcriptomic preprocessing and drug-response prediction workflow is summarized in Figure 1, while extended analyses, including pharmacogenomic filtering, prediction robustness, cohort comparisons, and prioritization of candidate compounds, are presented in Supplementary Figures S1-S4.

### Generation of Autologous APCs

Unrelated healthy blood donor PBMCs (partially HLA-matched with the pDMG patients) were isolated by Ficoll gradient (GE17-1440-03, Merk) and cryopreserved in Fetal Bovine Serum (FBS) (F9665-500ML, Merk) with 10% DMSO (D2650-100ml, Sigma). B cells were enriched using CD19 microbeads (Miltenyi Biotec, 130-050-301) and expanded with irradiated NIH 3T3-CD40L feeder cells (kindly provided by the laboratory of Dr. Steven Rosenberg, NCI, NIH) as described.^32^^,^^43^ Immature dendritic cells (DCs) were generated by plastic adherence followed by culture in RPMI-1640 + glutamax (72400-021, Thermofisher) supplemented with GM-CSF (800 UI/ml, Miltenyi Biotec, 130-093-867), IL-4 (200 U/ml, Miltenyi Biotec, 130-093-922), AB serum (10%, SigmaAldrich, H3667), for 5 days before cryopreservation.

### In Vitro Stimulation Assay

Neoantigen-reactive T cells screening were conducted as single replicates using an in vitro stimulation approach adapted from previously described CD137-based enrichment strategies for identifying mutation-reactive T cells.^31^ Briefly, healthy donor T cells were co-cultured with autologous antigen-presenting cells loaded with either predicted mutation-derived peptides or tandem minigenes (TMGs). This approach was designed to allow antigen processing and presentation by autologous APCs, followed by identification of antigen-activated T cells through induction of CD137 expression. For peptide-based stimulation, autologous CD40L-expanded B cells were pulsed with predicted neoantigen peptides at 10 µg/mL for 2 h, washed to remove excess peptide, and co-cultured with autologous T cells at 1 × 10^6^ cells/well. Cultures were maintained for two weeks to allow expansion of low-frequency antigen-reactive T cells. Where applicable, cytokine support consisting of IL-2 (1000 U/mL) and IL-21 (30 ng/mL) was added once weekly during culture to sustain T-cell viability and expansion, without cytokine supplementation during the final short-term restimulation step.

After the initial culture period, cells were restimulated for 24 h with peptide-pulsed autologous APCs before flow cytometry analysis of CD137 upregulation. In parallel, a TMG-based strategy was used to assess recognition of endogenously processed mutation-derived epitopes, following the principle described by Parkhurst et al.^31^ TMG constructs encoded up to seven selected hotspot pDMG mutations, including variants in PIK3CA, PTEN, and TP53 (Table S2). Synthetic TMG mRNA (GenScript, USA) was transfected into 5 × 10^5^ autologous dendritic cells using Lipofectamine 3000. After 24 h, transfected DCs were co-cultured with autologous T cells for two weeks, followed by 24 h restimulation with freshly transfected DCs prior to screening. Following restimulation, antigen-reactive T cells were identified by CD137 expression on viable CD3^+^ T cells, with CD4^+^ and CD8^+^ subsets analyzed separately. Responses were interpreted relative to negative control conditions, including T cells cultured with unloaded APCs, or APCs non-transfected control, depending on the assay format. Positive-control stimulation was included to confirm T-cell competence and CD137 detection, using plate-bound MACS GMP CD-3/OKT-3 1ug/mL (Miltenyi).

CD3^+^CD137^+^ T cells were subsequently isolated by flow cytometric sorting for downstream bulk TCR sequencing, enabling enrichment and characterization of candidate neoantigen-reactive clonotypes.

### Flow Cytometry Screening and Cell Sorting Prior to Bulk TCR Sequencing

Activated T cells were identified by CD137 expression.^33^^,^^34^ Cells were stained with CD3-BV650 (1: 50, Biolegend, 317324), CD4-BV711 (1: 50, Biolegend, 317440), CD8-BV570 (1: 50, Biolegend, 301038), CD137-PE (1: 50, BD, 555956) antibodies, and propidium iodide (Sigma-Aldrich, P4864), and analyzed after exclusion of dead cells. HLA-A2 expression was assessed on GBMC6 (kindly provided by the laboratory of Dr. Massimo Dominici, University of Modena and Reggio Emilia, Italy), Huh-7 (kindly provided by the laboratory of Dr. Volker Lohmann, Heidelberg University, Germany),^35^ and DCs using fluorochrome-conjugated anti–HLA-A2-PE-Cy7 antibody (1: 100, 561347 BD).

For sorting, CD3^+^CD137^+^ T cells were isolated on a BD FACS Aria Fusion into lysis buffer. RNA was extracted and used for bulk TCR sequencing by a 5′-RACE-based protocol on the Illumina MiSeq (600-cycle kit). Reads were processed with FastQC, trimmed, aligned to TCR clonotypes, and assembled for CDR3 and full-length sequence analysis. A positive control consisting of multiple CDR3 clonotype peaks spanning a range of fragment lengths was included to support sequencing performance assessment and broad clonotype representation.

### Statistical Analysis and Visualization

Statistical analyses were performed in R and GraphPad Prism. Differences in neoantigen burden and T-cell responses were assessed using the Mann-Whitney U test, with *P *< .05 considered significant. Data wrangling and formatting were performed using the data.table and string packages in R. For broad comparisons across groups, imputed DSS values were categorized by specific MOLECULAR_SUBTYPE, CANCER_TYPE, and CANCER_GROUP using clinical annotations provided by OpenPBTA. Distributional differences of DSS across these subtypes were visualized as boxplots using ggplot2. To visualize broad pharmacological response profiles across the pDMG cohort and normal/reference tissues, row-wise Z-scoring was applied to the imputed DSS values to highlight sample-specific relative sensitivities for each compound. Heatmaps of the Z-scored DSS matrix, incorporating hierarchical clustering (Euclidean distance) of both samples and compounds, were generated using the ComplexHeatmap package.^44^ Custom color annotations were applied to classify compounds by mechanistic target families (eg, kinase inhibitors, topoisomerase inhibitors, receptor agonists).

## Results

### Patient Characteristics

A retrospective clinical analysis was performed on a historical cohort of 6 pediatric patients diagnosed with diffuse intrinsic pontine glioma (pDMG). The cohort consisted of 3 female and 3 male patients, all of whom were deceased at the time of data analysis, details are reported in Table 1. The age at death ranged from 5 to 8 years. Histopathological evaluation revealed that 5 of the 6 patients were diagnosed with diffuse midline glioma harboring the H3K27M mutation, consistent with the World Health Organization (WHO) grade IV classification. One patient was diagnosed with a cell-rich, highly malignant glioma exhibiting glial differentiation, also graded as WHO grade IV. Treatment regimens varied among patients but consistently included radiotherapy, which was administered in 5 out of 6 cases. Targeted therapies were used in combination with radiotherapy, including Erlotinib (administered to 3 patients), Everolimus (administered to 3 patients), Dasatinib, and Bevacizumab. No details were available for one patient (DIPG4), since treatment had been performed outside Sweden. Overall survival (OS) ranged from 6 to 18 months, with a median OS of approximately 9 months. The longest survival was observed in a patient who received Erlotinib in combination with radiotherapy (18 months), while the shortest OS (6 months) was reported in two patients: one treated with Erlotinib and radiotherapy, the other one with Everolimus and radiotherapy. Clinical notes indicated that one patient presented with a branchio-oculo-facial syndrome while no additional syndromic features or comorbidities were documented for the remaining patients.

**Table 1.** Clinical, pathological, and molecular characteristics of pediatric patients diagnosed with ponsgliomas

Sample ID	Diagnosis	Clinical information	Treatment	OS (months)
DIPG1	pDMG *H3K27*-altered, grade IV	female, dead at age of 7, brachofacial syndrome	Erlotinib, Radiotherapy proton	18
DIPG3	Ponsglioma	male, dead at age of 7	Erlotinib, Radiotherapy proton	6
DIPG4	Ponsglioma. Cell-rich highly malignant tumor with glial differentiation, histological, grade IV	female, dead at age of 5	No data available	8
DIPG7	pDMG *H3K27*-altered, grade IV	female, dead at age of 7	Radiotherapy, Everolimus, Dasatinib, Erlotinib, Bevacizumab	14
DIPG8	pDMG *H3K27*-altered, grade IV	male, dead at age of 6	Radiotherapy, Everolimus	9
DIPG9	pDMG *H3K27*-altered, grade IV	male, dead at age of 8	Everolimus, Radiotherapy	6

### Mutational Landscape of pDMG Tumor Samples

The genetic characteristics of pDMG tumor samples were assessed to support neoantigen discovery and evaluate the performance of the Personalized Immuno-Oncology Ranking (PIOR) platform for comprehensive molecular characterization. PIOR enabled management and analysis of the large WGS and RNA-seq datasets and facilitated identification of variants relevant for neoantigen prediction. After quality control of DNA and RNA sequencing data, variants from each tumor were analyzed using matched normal WGS (blood), tumor WGS, and tumor RNA-seq libraries. The platform ranked candidate variants based on a weighted algorithm incorporating 53 parameters, from which the top 1000 were selected for manual review. Curated variants were visually confirmed using BAM coverage through the integrated JBrowser tool. The number of validated variants per tumor and their correspondence with histological and pathological features are summarized in Supplementary Table S3.

Comprehensive genomic profiling of the pDMG cohort revealed substantial heterogeneity in mutational burden, gene expression, and predicted therapeutic relevance (Supplementary Table S4). Mutation counts varied widely, from 6 in DIPG1 to 15 in DIPG8, indicating diverse levels of genomic instability across tumors. Expression of mutated genes, assessed by total RNA coverage (TotCov_RNA) and alternative allele coverage (Cov_Alt_RNA), also differed markedly. H3-3A mutations showed the highest expression across samples, reaching TotCov_RNA 2748 and Cov_Alt_RNA 1489 in DIPG3. Other highly expressed variants included AL354733.3 in DIPG4 (TotCov_RNA 903, Cov_Alt_RNA 407), TUFM in DIPG7 (TotCov_RNA 598, Cov_Alt_RNA 46), and PIK3R1 in DIPG1 (TotCov_RNA 366, Cov_Alt_RNA 161). Most variants (85.7%) were Single Nucleotide Variants (SNVs), with fewer deletions (8.6%) and insertions (1.4%), consistent with mutational processes often observed in tumors with impaired DNA damage repair such as TP53-mutant pDMG. Variant scores ranged from 15.9 to 76.5 (median 41.4). High-scoring variants included TP53 in DIPG4 (76.5), ACVR1 in DIPG1 (68.2), AL354733.3 in DIPG4 (68.6), and TP53 in DIPG8 (66.2), and these generally correlated with higher expression levels, suggesting functional relevance. Predicted HLA-binding analysis showed that ∼45.7% of mutations were likely to be presented as neoantigens. All H3-3A and TP53 mutations demonstrated HLA-binding potential, marking them as strong immunotherapeutic candidates. DIPG3 and DIPG8 exhibited the highest proportion of mutations with predicted immunogenicity. Subcellular localization analysis distinguished intracellular from plasma membrane–associated targets. About 18.6% of mutated genes encoded membrane proteins, including *PLXNA3*, *ITGAX*, *KCNU1*, *COL4A5*, *CD3G*, *POLE*, and *SLC35G2*, representing candidates for antibody- or CAR-T-based therapies. DIPG9 contained the highest fraction of membrane-localized variants. The remaining mutations, including *TP53*, *PIK3CA*, *H3F3A*, and *ACVR1*, were intracellular and detected across six samples. Although not accessible to antibody- or CAR-T–mediated recognition, these intracellular proteins generate peptides presented on MHC molecules, making them suitable targets for TCR-T cell therapy and expanding the immunotherapeutic landscape for pDMG.

### Transcriptional Drug Response Profiling in pDMG

To explore potential therapeutic strategies in this cohort, tumor mRNA expression profiles from each patient were analyzed using oncoPredict^41^ in conjunction with the Genomics of Drug Sensitivity in Cancer (GDSC) database^45^ and presented in Figure 1. This evaluation is intended as hypothesis generation tool and not as validated predictive model, we also performed the same analysis on the larger public database (Open Pediatric Brain Tumor Atlas, OpenPBTA^38^) for the reader to have a reference, the results are displayed in Supplementary Figures S1-S4. In brief, predictive models were trained to infer observed IC50 values across a large panel of compounds tested in cancer cell lines, and these models were subsequently applied to RNA-seq profiles derived from pDMG patient samples. This approach identified candidate vulnerabilities that may support the exploitation of H3K27M-associated defects through synthetic lethality and may support future stratification within the pDMG spectrum. As shown in Figure 1, the model predicted high IC50 sensitivity for the pan-HDAC inhibitor panobinostat, a compound that has been extensively investigated as a first-line therapeutic option in pDMG. Panobinostat is particularly attractive for CNS malignancies due to its demonstrated blood-brain barrier penetrance^46^ and favorable safety profile in clinical trials.^47^ Mechanistically, this predicted sensitivity is consistent with a synthetic lethal interaction: the H3K27M oncohistone impairs PRC2 function and reduces H3K27 trimethylation, thereby increasing cellular dependence on histone deacetylase activity to maintain chromatin stability.^48^ In addition, the screen highlighted several microtubule-destabilizing agents and kinesin spindle protein inhibitors (annotated in green as “Microtubule” in Figure 1). Although these findings are biologically relevant as H3K27M has been shown to disrupt chromosome organization during mitosis,^49^ suggesting a potential synthetic lethal vulnerability when mitotic integrity is further compromised, they require further functional and clinical validation. Identified compounds included vinorelbine, vinblastine, docetaxel, paclitaxel, AZD4877, ARRY-520, and notably epothilone B (patupilone). Among these, epothilone B demonstrated the strongest and most consistent IC50 predictions and is distinguished by its superior CNS bioavailability, documented antineoplastic activity in glioma models,^50^ and efficient blood-brain barrier penetration.^46^ The analysis also revealed substantial inter-patient heterogeneity in predicted drug sensitivity. For example, rapamycin (used here as a functional proxy for the mTOR inhibitor Everolimus) was predicted to be selectively effective in DIPG1 and DIPG3. Conversely, several patient-drug combinations showed reduced predicted sensitivity, such as DIPG8 to dinaciclib, DIPG4 to daporinad, and DIPG9 to sepantronium bromide. Erlotinib and dasatinib were likewise predicted to have minimal efficacy across the analyzed cases, despite their prior clinical use in several patients. Collectively, these findings underscore the limitations of a “one-size-fits-all” treatment paradigm and strongly support the incorporation of RNA sequencing as a companion diagnostic to guide patient stratification and prioritize therapies based on individual molecular vulnerabilities.

### Assessment of Immunotherapy Targets in pDMG: Surface Antigens

Given the limited efficacy of pharmacological inhibitors in pDMG, immunotherapy has become an increasingly attractive therapeutic strategy. CAR-T cell approaches targeting surface antigens such as GD2 have shown promising safety and early clinical activity in pediatric patients with H3K27M-mutant diffuse midline gliomas.^29^^,^^51^^,^^52^ GD2 expression in brain tumors is driven by B4GALNT1 (GM2/GD2 synthase), the enzyme converting GD3 to GD2. Elevated B4GALNT1 activity correlates with increased GD2 surface levels in neuroectodermal tumors, including diffuse midline gliomas,^53^ providing a strong molecular rationale for GD2-directed CAR-T therapies in pDMG.

**Figure 2.**
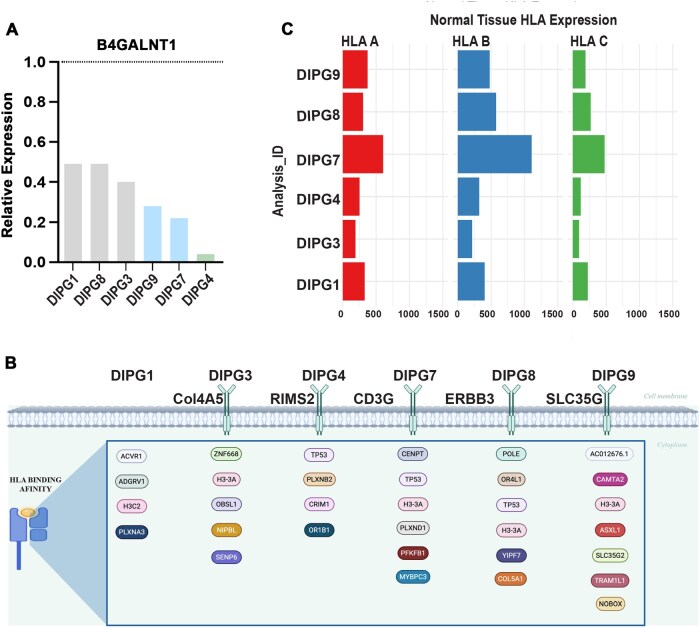
B4GALNT1 expression and intracellular HLA-presented targets in pDMG. (A) Bar plot of B4GALNT1 (GM2/GD2 synthase) mRNA levels across the cohort. Each bar represents one pDMG sample; GBMC6 is included as a technical reference for validated GD2 expression. (B) Schematic of predicted intracellular targets by sample. Genes shown inside the cytoplasmic compartment correspond to peptides with high predicted HLA class I binding affinity given each tumor’s HLA type. Sample-specific cell-surface candidates are annotated at the cell membrane compartment. Schematic representation made using biorender https://app.biorender.com/illustrations/689c83a85c0d0f31f0f15c2d. (C) Expression levels of HLA-A, HLA-B, and HLA-C genes shown as reads per million (RPM). Leukocytes were added as control.

To assess the relevance of GD2-targeted immunotherapy in our cohort, we quantified B4GALNT1 expression in pDMG samples utilizing as a technical positive reference GBMC6, a glioblastoma-derived cell line with validated GD2 expression and established recognition by GD2-specific T cells in vitro and in vivo.^54^ Based on expression ratios relative to GBMC6, samples were grouped into high, intermediate, and low GD2-expressing categories (Figure 2A), however, this categorization has the only purpose of comparison with a known sample, it does not constitute a predictive data for sensitivity to anti-GD2 therapy. DIPG1 and DIPG8 showed the highest B4GALNT1 expression (ratio 0.49), followed by DIPG3 (0.40), indicating substantial GD2 biosynthetic activity, DIPG9 (0.28) and DIPG7 (0.22) fell into the intermediate category, while DIPG4 demonstrated minimal expression (0.04). Because tumor cellularity could influence transcript abundance, we compared samples with similar tumor content (DIPG3: 30%-40%; DIPG4: 25%; Table S3). No meaningful differences attributable to tumor fraction were observed, indicating that B4GALNT1 expression levels reflect true biological variation rather than sampling bias.

### Assessment of Immunotherapy Targets in pDMG: Neoantigens

Building on the rationale for CAR-T targeting of surface antigens such as GD2, we next examined intracellularly altered genes in pDMG to assess their suitability for TCR-based immunotherapy. Unlike CAR-T cells, which require membrane-bound antigens, TCR-T cells can target peptides derived from intracellular oncogenic drivers once presented by HLA molecules. HLA-binding predictions confirmed that several pDMG mutations generate high-affinity ligands, supporting their potential for antigen presentation (Supplementary Table S4). Among these, the *H3K27M* (H3-3A) mutation, *TP53* alterations, and *ACVR1* mutations emerged as key intracellular oncogenic drivers consistently predicted to form strong HLA-binding peptides. Sample-level analysis showed that biologically relevant mutations, *ACVR1* in DIPG1, *H3K27M* in DIPG3, DIPG7, and DIPG9, *TP53* in DIPG4, DIPG7, and DIPG8, and *POLE* in DIPG8, are predominantly intracellular and predicted to produce high-affinity neoepitopes (Figure 2B). These findings underscore the importance of TCR-based approaches, as these core pDMG drivers are not targetable by CAR-T strategies restricted to surface antigens. We also identified a smaller set of cell surface proteins with predicted mutations in specific samples, including *COL4A5* (DIPG3), PIMREG (DIPG4), ERRFI1 (DIPG8), and *SLC35G2* (DIPG9). Although less recurrent than intracellular alterations such as *H3K27M* or *TP53*, their plasma membrane localization makes them feasible targets for antibody-based or CAR-T therapies. Together, these findings highlight a dual immunotherapeutic landscape in pDMG: CAR-T cells are suited for the limited set of membrane-bound antigens and TCR-T cells can expand targetability to the more frequent and biologically critical intracellular oncogenic drivers presented on HLA complexes. This complementary targeting strategy may be necessary to address the heterogeneous molecular architecture of pDMG.

### HLA Expression and Immune Cell Composition Across pDMG Samples

To evaluate the feasibility of targeting neoantigens via TCRs, we assessed HLA expression in pDMG samples, a critical factor for antigen presentation in brain tumors (Supplementary Table S5). HLA typing and peptide-HLA predictions identified multiple high-affinity class I binders, including *H3K27M*-, *TP53*-, and *ACVR1*-derived peptides, suggesting antigen processing and presentation are largely preserved. Analysis of HLA class I alleles revealed a diverse repertoire, with frequent A*02:01, A*24:02, and B*44:02 alleles. DIPG1, DIPG4, and DIPG9 shared A*02:01, while DIPG3 carried A*24:02, reflecting recurrent genotypes relevant for peptide-HLA binding. Additionally, alleles associated with strong antigen presentation, such as B*07:02 (DIPG1) and C*07:02 (DIPG1, DIPG3, DIPG9), were also detected.

**Figure 3.**
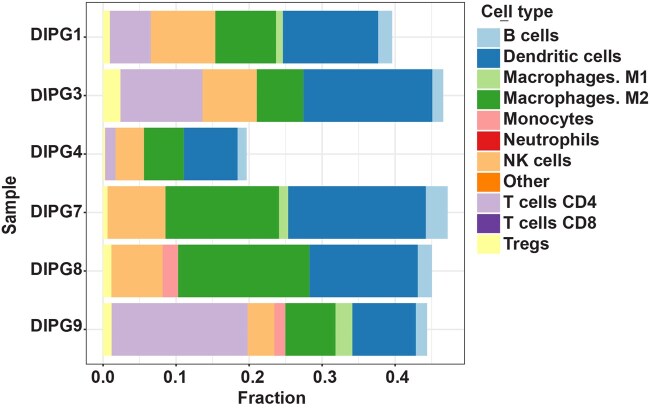
Immune cell composition across pDMG and GBM samples. Relative immune cell fractions inferred from transcriptomic data in pDMG and GBM samples, including B cells, dendritic cells, macrophages (M1, M2), monocytes, neutrophils, NK cells, CD4^+^ T cells, CD8^+^ T cells, and Tregs.

HLA class I genes (HLA-A, -B, -C) were expressed across pDMG samples (Figure 2C), confirming transcriptional activity. Transcriptomic analysis (RPM-normalized) revealed variable expression: DIPG7 showed the highest HLA-A (500 RPM) and HLA-B (1200 RPM), followed by DIPG1 and DIPG8, while DIPG3, DIPG4, and DIPG9 had intermediate or lower levels. HLA-C expression mirrored HLA-B patterns but was generally lower. GBMC6 glioblastoma exhibited the strongest HLA expression, reflecting higher tumor cellularity, and served as a reference. Flow cytometry confirmed surface HLA-A2 on GBMC6, aligning with transcriptional data and A*02:01 typing (Supplementary Figure S5A), validating potential TCR recognition. Compared with normal tissues (Supplementary Figure S5B), pDMG HLA levels were lower than immune-enriched organs but similar to normal brain, except DIPG7, which showed elevated HLA expression, suggesting localized immune activation. Immune cell analysis (Figure 3) indicated 25%-45% infiltration across six pDMG samples, with DIPG4 lowest and DIPG3, DIPG7, and DIPG8 highest. Dendritic cells comprised 10%-15% of total cells, particularly enriched in DIPG3, DIPG7, and DIPG8, supporting retained antigen presentation. Macrophages were abundant, skewed toward immunosuppressive M2 phenotypes (15%-20% in DIPG7/8), while M1 macrophages were low (max 5% in DIPG9). T cells were predominantly CD4^+^ (10%-15% in DIPG1, 3, 9), with scarce CD8^+^ T cells and low Tregs (2%-3%), indicating an immunosuppressive environment. B cells (≈5%) and NK cells (5%-10%) were present across samples, with DIPG1, 3, and 9 showing higher NK frequencies. Monocytes were enriched in DIPG8 and DIPG9, reflecting variable myeloid recruitment.

### Pipeline Combination Improves Prediction Performance

Given the heterogeneity in immune infiltration, antigen presentation, and tumor-specific targets across pDMG samples, we enhanced neoantigen evaluation by integrating multiple prediction algorithms. Candidate neoantigens were prioritized using a combined computational pipeline (PIOR plus Ardigen) and assessed for functional immunogenicity via in vitro stimulation assays to identify peptides capable of eliciting T cell responses. Six pDMG samples were analyzed for HLA binding and short peptide prediction, revealing distinct SNP profiles (Table S6). Some variants were shared, eg, DIPG3 and DIPG9 exhibited similar SNPs on chromosomes 2, 6, and 7, while recurrent SNPs on chromosomes 2 and 6 suggested mutational hotspots. Unique variants were detected, such as chr22:50280523: C: T in DIPG4 and variants on chromosomes 1, 16, and 20 in DIPG9, not identified by PIOR, indicating potential driver mutations. DIPG7 consistently displayed SNPs on chromosomes 7 and 16, highlighting their potential role in antigenicity and immune interactions. Integrating the two pipelines provided a more comprehensive analysis, yielding robust predictions of biologically relevant peptides. This approach enabled selection of short peptides for in vitro assays, supporting functional evaluation of neoantigen immunogenicity and advancing pDMG immunotherapy research.

### Immunological Screening for T Cell Reactivity to Selected Mutations

**Figure 4.**
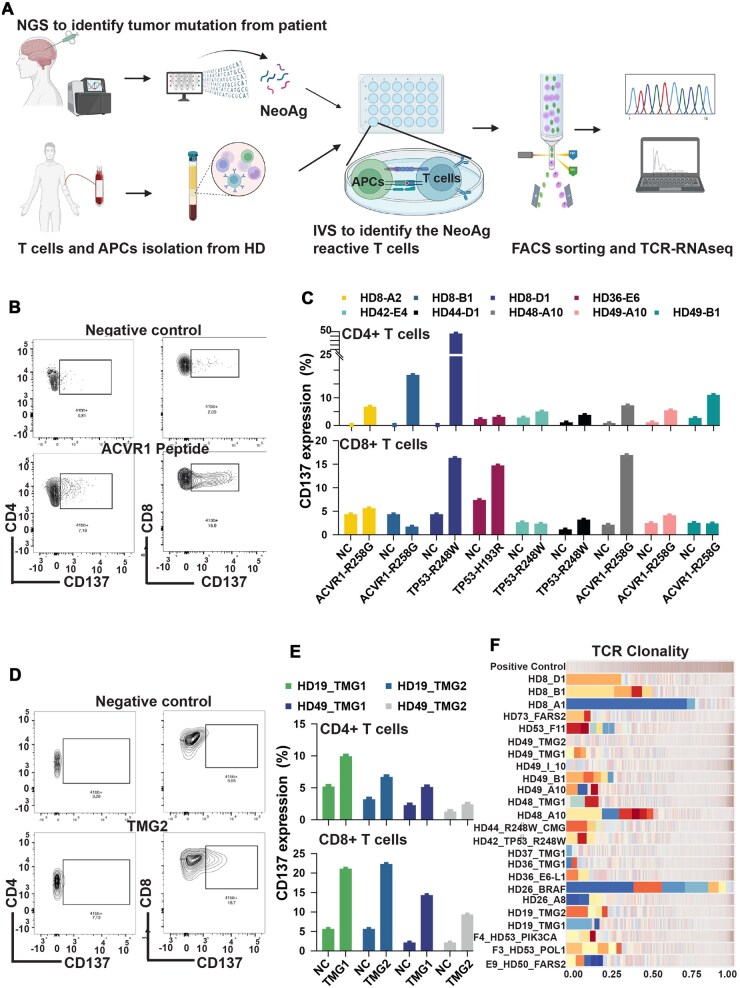
Immunological screening and TCR repertoire analysis of neoantigen-reactive T cells. (A) Schematic overview of the experimental workflow. PBMCs from unrelated but partially HLA-matched healthy donors were stimulated in vitro with TMGs or single peptides encoding neoantigens using autologous APCs. After 2 weeks of culture, antigen-reactive T cells were identified by upregulation of CD137 and sorted for TCR sequencing. Samples were considered positive when the frequency of CD137^+^ T cells was at least two-fold higher than that observed in the negative control condition (NC), consisting of T cells co-cultured with APCs unloaded with peptides or non-transfected with TMG. A positive control using plate-bound anti-CD3 stimulation was included to confirm CD137 upregulation in activated T cells assessed by FACS. (B) Representative flow cytometry plots showing CD137 expression on CD4^+^ and CD8^+^ T cells after stimulation with selected peptides encoding neoantigens. (C) Quantification of CD137^+^ T cell responses in CD4^+^ and CD8^+^ subsets across multiple donors and neoantigens. Bars represent the proportion of reactive T cells among live singlets. (D) Representative flow cytometry plots showing CD137 expression on CD4^+^ and CD8^+^ T cells after stimulation with selected tandem mini genes 1 or 2 (TMG1 or TMG2). (E) Quantification of CD137^+^ T cell responses in CD4^+^ and CD8^+^ subsets across multiple donors and tandem mini genes. Bars represent the proportion of reactive T cells among live singlets. (F) Clonal composition of CD137^+^ T cell populations assessed by TCR sequencing. Expanded clonotypes were detected in response to stimulation by peptides or TMGs, indicating oligoclonal expansion. Each bar represents the relative TCR Vβ frequency distribution of TCR clonotypes within individual samples. Positive control with broad CDR3 clonotype distribution across multiple fragment lengths was used to assess sequencing performance.

To evaluate T cell reactivity against HLA-predicted peptides and TMGs, APCs were isolated from healthy donors for co-culture screenings. Due to limited T cell availability in pediatric pDMG patientsoften reduced by steroid treatment, a proof-of-principle approach was adopted. This involved mimicking potential outcomes using HLA-matched samples from unrelated donors, an alternative, conceptually similar approach, could have been to use APCs from the patient’s parents. The screening workflow is summarized in Figure 4A. Coding variants from missense mutations in pDMG tumors (from targeted and whole-exome sequencing) were manually curated, focusing on SNVs. Short peptides predicted to bind HLA, derived from shared, driver, or frequently mutated pDMG genes, were selected for immunogenicity assays. Among 22 healthy donors tested, putative T cell reactivity was detected in 6 donors stimulated with short peptides and in 2 donors stimulated with TMGs (Table 2).

**Table 2.** Reactivity of healthy donor T cells against HLA predicted short peptides and tandem minigenes

Healthy donor ID	HLA typing	Unique sample ID	Target	Reactive T cells
HD8	A 02:01/30:01	HD8-A2	ACVR1 p. R258G	CD4
	B 13:02/44:02	HD8-B1	ACVR1 p. R258G	CD4
	C 05:01/06:02	HD8-D1	TP53 p. R248W	CD4 and CD8
HD36	A 03:01/68:01 B 15:01/27:05 C 03:04/07:04	HD36-E6	TP53 p. H193R	CD8
HD42	A 02:01/68:01	HD42-E4	TP53 p. R248W	CD4
	B 07:02/44:02			
	C 05:01/07:02			
HD44	A 02:01/02:08	HD44-D1	TP53 p. R248W	CD4 and CD8
	B 18:01/51:01			
	C 04:01/12:03			
HD48	A 02:01/11:01	HD48-A10	ACVR1 p. R258G	CD4 and CD8
	B 44:02/51:01			
	C 03:03/05:01			
HD49	A 02:01/23:01 B 15:01/44:03 C 03:04/04:01	HD49-A10	ACVR1 p. R258G	CD4
		HD49-B1	ACVR1 p. R258G	CD4
HD19	A 01:01/23:01 B 13:02/44:03 C04:01/06:02	HD 19_TMG1	TMG1	CD8
		HD 19_TMG2	TMG2	CD8
HD49	A 02:01/23:01 B 15:01/44:03 C 03:04/04:01	HD 49_TMG1	TMG1	CD8
		HD 49_TMG2	TMG2	CD8

T cells from multiple healthy donors were screened for CD137 upregulation following stimulation with predicted neoantigens, either as peptides or TMGs. CD137 expression and gating strategies are shown in Figure 4B-E. Reactivity was donor- and mutation-dependent, involving both CD4^+^ and CD8^+^ T cells. Samples were considered positive when the frequency of CD137^+^ T cells was at least two-fold higher than that observed in the negative control condition, consisting of T cells co-cultured with APCs unloaded with peptides or TMG. A positive control using plate-bound anti-CD3 stimulation was included to confirm CD137 upregulation in activated T cells assessed by FACS. Peptides encoding *ACVR1*_R258G consistently induced CD4^+^ responses across three donors, with HD48 also activating CD8^+^ T cells, indicating broader immunogenicity. TP53 mutations elicited variable responses: R248W activated both CD4^+^ and CD8^+^ T cells in some donors, whereas H193R triggered CD8^+^ T cells specifically, highlighting subset-specific recognition patterns (Figure 4B and C). Overall, ACVR1 mutations predominantly engaged CD4^+^ T cells, whereas TP53 mutations activated both subsets, revealing a spectrum of immunogenic epitopes. TMGs encoding hotspot mutations (*TP53*, *PTEN*, *PIK3CA*) induced CD8^+^ T cell activation but not CD4^+^ responses. In HD19, both TMG1 and TMG2 activated CD8^+^ T cells, and in HD49, either TMG triggered CD137^+^ CD8^+^ expansion, demonstrating that distinct TMG-encoded epitopes can stimulate overlapping CD8^+^ repertoires (Figure 4D and E).

### TCR Repertoire Analysis of CD137+ T Cells Reactive to pDMG Mutation-Derived Peptides

TCR repertoire analysis provides insight into the specificity and diversity of T-cell responses against tumor antigens. We evaluated TCR clonality in CD137^+^ T cells sorted after stimulation with predicted HLA-binding peptides from pDMG samples. These CD137^+^ T cells, putatively reactive to the antigens, were enriched and subjected to bulk TCR RNA-seq to characterize clonotype diversity (TRA, TRB, TRD, TRG CDR3 regions). Bulk TCR sequencing revealed donor- and antigen-dependent differences in beta chain expansion (Figure 4F). Peptide stimulation in HD8_A2, HD8_B1, and HD8_D1 drove strong expansion of dominant clonotypes, indicating robust putative reactivity, whereas HD36_E6 and HD42_E4 responses were more polyclonal. Moderate clonal enrichment was seen in HD44_D1, and limited expansion in HD48_A10 and HD49_A10, with HD49_B1 showing stronger enrichment similar to HD8. TMG stimulations (HD19_TMG1/2, HD49_TMG1/2) elicited clonal responses with distinct repertoire patterns, indicating engagement of overlapping but non-identical T cell subsets. These results highlight that some epitopes drive strong, focused clonal expansion, while others induce moderate or diverse responses. Clonality analysis thus enables functional stratification of putatively reactive peptide-specific TCRs, guiding selection of promising immunogenic targets.

## Discussion

pDMG remains one of the most therapeutically challenging pediatric malignancies, with limited benefit from conventional treatment modalities and a continued need for biologically informed therapeutic strategies. In this study, we integrated genomic, transcriptomic, and immunogenomic analyses of a rare Swedish cohort of primary pDMG samples to identify candidate molecular vulnerabilities, surface-antigen expression patterns, and mutation-derived neoantigens with potential relevance for adoptive cellular and gene-based immunotherapy. Consistent with the known clinical course of pDMG, the patients in this cohort experienced poor outcomes despite treatment with radiotherapy and, in several cases, targeted or systemic agents. The heterogeneity in treatment regimens, including use of Erlotinib, Everolimus, Dasatinib, and Bevacizumab, reflects the absence of a uniformly effective systemic treatment strategy for newly diagnosed pDMG. Previous clinical studies, including BIOMEDE 1.0 and HERBY, have similarly highlighted both the feasibility of biomarker-guided trials and the limited survival benefit achieved by currently available targeted or anti-angiogenic approaches.^55-58^ These observations underscore the need for complementary strategies that incorporate molecular profiling, immune-target discovery, and functional validation. Our transcriptome-based drug-response profiling identified heterogeneous predicted sensitivities across the analyzed tumors. In particular, the analysis highlighted candidate vulnerabilities involving HDAC inhibition, microtubule-targeting agents, and mitotic pathway disruption. These findings are biologically plausible in the context of H3K27M-driven chromatin dysregulation and altered mitotic control, but they should be interpreted as hypothesis-generating. Although the analysis recovered agents previously explored in pDMG and suggested additional candidates such as microtubule-destabilizing compounds, these predictions were not directly validated in patient-derived functional models. Therefore, they provide a rationale for future preclinical testing rather than evidence of clinical efficacy or immediate treatment stratification. Our analysis of immunotherapy targets further supports the concept that pDMG may require individualized or combinatorial targeting approaches. B4GALNT1 transcript levels varied across samples, suggesting inter-tumoral heterogeneity in the GD2 biosynthetic pathway. Because GBMC6 was used only as a technical reference with validated GD2 expression, these transcript-level comparisons should not be interpreted as definitive evidence of GD2 surface abundance or sensitivity to GD2-directed CAR-T therapy. Nevertheless, the observed variability is consistent with the broader concept that antigen heterogeneity may influence suitability for CAR-T approaches. Confirmation at the protein and cell-surface level will be required to determine whether B4GALNT1 expression reliably predicts GD2-targetability in this setting. In parallel, the genomic and neoantigen analyses identified recurrent intracellular alterations, including ACVR1, TP53, and H3K27M-associated variants, that may be relevant for TCR-based immunotherapy if processed and presented by HLA molecules. Several predicted mutation-derived peptides showed favorable HLA-binding features, supporting their prioritization for functional testing. Importantly, intracellular oncogenic drivers are not accessible to antibody- or CAR-based recognition but may be targetable through peptide-HLA recognition by TCR-engineered T cells. This highlights the potential complementarity of CAR-T and TCR-T strategies in pDMG, where surface-antigen availability may be limited and heterogeneous, while intracellular driver mutations may provide additional immunotherapeutic opportunities. The immune-inference analysis suggested a microenvironment characterized by variable immune infiltration, enrichment of myeloid populations, and relatively low cytotoxic T-cell and NK-cell signals. These findings are consistent with an immunologically constrained or suppressive pDMG microenvironment. However, because the deconvolution approach used here is based on bulk RNA-seq and is optimized for immune-cell inference, it does not robustly quantify tumor, stromal, or endothelial compartments. Therefore, these results should be interpreted as transcript-based estimates of immune composition rather than a comprehensive cellular map of the tumor microenvironment. Orthogonal validation using spatial, histological, or single-cell approaches would be needed to define the cellular architecture more precisely. A key component of this study was the proof-of-principle functional screening of predicted neoantigens using unrelated, partially HLA-matched healthy donor T cells. CD137 upregulation after peptide or tandem-minigene stimulation identified candidate antigen-reactive T-cell populations, and subsequent TCR sequencing revealed clonotype enrichment in selected conditions. These findings suggest that some pDMG-associated mutations can elicit detectable T-cell activation in vitro. However, the observed responses were donor- and target-dependent, which is expected given differences in HLA restriction, precursor frequency, and individual TCR repertoires. Thus, these data should be viewed as an initial functional prioritization strategy rather than definitive validation of therapeutic TCRs. Further studies will be required to confirm antigen specificity, HLA restriction, functional avidity, tumor recognition, and safety of candidate TCRs. Notably, not all recurrent pDMG mutations are necessarily suitable immunotherapy targets. For example, prior work has shown that H3.3K27M may be poorly presented in some HLA contexts, emphasizing that mutation recurrence alone is insufficient to define an actionable T-cell target.^59^ Our workflow addresses this challenge by combining genomic identification, HLA-binding prediction, CD137-based functional screening, and TCR repertoire analysis. This integrated approach may help prioritize neoantigens that are not only predicted computationally but also capable of inducing measurable T-cell activation. Nonetheless, additional validation will be essential before any candidate antigen or TCR can be considered for therapeutic development. Several limitations should be acknowledged. First, the cohort size is small and retrospective, and the findings should therefore be interpreted as discovery-oriented rather than prevalence-defining. Second, drug-response predictions were inferred from transcriptomic data and were not directly validated in patient-derived functional assays; these results should therefore be considered hypothesis-generating. Third, transcript-based antigen prioritization, including B4GALNT1 expression, was not systematically validated at the protein or cell-surface level because of limited available material. Fourth, immune-cell composition was inferred from bulk RNA-seq and does not capture spatial organization or non-immune cellular compartments. Finally, although CD137-based enrichment identified candidate neoantigen-reactive T-cell populations and clonotypes, these findings require further validation of antigen specificity, HLA restriction, tumor recognition, and functional activity before therapeutic translation. In summary, this study provides an integrated molecular and immunogenomic characterization of rare primary pDMG samples and identifies candidate therapeutic vulnerabilities, variable surface-antigen expression patterns, and putative neoantigen-reactive T-cell responses. The findings support the feasibility of combining genomic, transcriptomic, and functional immune-screening approaches to guide future development of personalized cellular and gene-based therapies for pDMG. While the results remain exploratory, they provide a framework for prioritizing candidate targets for further preclinical validation and for designing future combinatorial strategies that address the molecular heterogeneity and immunosuppressive biology of this devastating pediatric tumor.

## Supplementary Material

vdag193_Supplementary_Data

## Data Availability

The data will be made available upon request by contacting the Barntumorbanken/Swedish Childhood Tumor Bank after ethical approval.
