# Supplementary material for: Immunogenomic profiling reveals targets for gene therapy in pediatric brain tumors
Source: Neurooncol Adv. 2026 Jul 27;8(1):vdag193. doi: 10.1093/noajnl/vdag193 (PMC13464507; doi:10.1093/noajnl/vdag193)
Supplement: Supplementary file 1 [file vdag193_supplementary_data.zip › Supplementary tables and figure legends.docx]

**Table S1: Short peptides from HLA binding prediction and corresponding amino acids sequences**

| **Gene** | **Mutation** | **HLA binding** | **AA sequence** |
| --- | --- | --- | --- |
| *ACVR1* | R258G | A02 | MLGHENILGFI |
| *ACVR1* | R258G | A02 | VMLGHENIL |
| *H3F3A* | K28M | B27 | ARMSAPSTGGV |
| *H3F3A* | K28M | C03 | MSAPSTGGV |
| *H3F3A* | K28M | A03 | MSAPSTGGVK |
| *H3F3A* | K28M | A03 | RMSAPSTGGVK |
| *HIST1H3B* | K28M | B07 | AARMSAPAT |
| *HIST1H3B* | K28M | A02 | RMSAPATGGV |
| *TP53* | R248W | A02 | CMGGMNWRPI |
| *TP53* | R248W | A02 | GMNWRPILTI |
| *TP53* | R158G | C12 | GTRVGAMAI |
| *TP53* | H193R | A03 | RLIRVEGNLR |

**Table S2: TMG IDs with gene mutations and amino acid Sequences**

| **TMG ID** | **Gene mutation** | **Amino acid sequence** |
| --- | --- | --- |
| TMG1 | KRAS p.G12D | MTEYKLVVVGADGVGKSALTIQLI |
|  | KRAS p.G12V | MTEYKLVVVGAVGVGKSALTIQLI |
|  | PIK3CA p.E542K | QLKAISTRDPLSKITEQEKDFLWSH |
|  | PIK3CA p.E545A | AISTRDPLSEITAQEKDFLWSHRHY |
|  | PIK3CA p.E545K | AISTRDPLSEITKQEKDFLWSHRHY |
|  | PIK3CA p.H1047R | ALEYFMKQMNDARHGGWTTKMDWIF |
|  | PIK3CA p.R88Q | EAEREEFFDETRQLCDLRLFQPFLK |
| TMG2 | PTEN p.R173C | RDKKGVTIPSQRCYVYYYSYLLKNH |
|  | TP53 p.R175H | YKQSQHMTEVVRHCPHHERCSDSDG |
|  | TP53 p.R248Q | YMCNSSCMGGMNQRPILTIITLEDS |
|  | TP53 p.R248W | YMCNSSCMGGMNWRPILTIITLEDS |
|  | TP53 p.R249S | MCNSSCMGGMNRSPILTIITLEDSS |
|  | TP53 p.R273C | SGNLLGRNSFEVCVCACPGRDRRTE |
|  | TP53 p.V157F | LWVDSTPPPGTRFRAMAIYKQSQHM |

Each minigene is 25 amino acids long, centered on the mutation (red marked) with 12 wild-type amino acids on each flank.

**Table S3. Pathology reports, sequencing libraries, and approved variants in pDMG samples**

| Sample ID | Pathology report | Tumor sequencing library (DNA) | Tumor sequencing library (RNA) | Blood sequencing library (DNA) | Approved variants |
| --- | --- | --- | --- | --- | --- |
| DIPG1 | pDMG grade IV, H3 K27M-mutated. > 70% tumor cells. | WGS (90X) | RNASeq (130 M reads) | WGS (30X) | 7 |
| DIPG3 | Ponsglioma. 30-40% tumor cells. | WGS (90X) | RNASeq (100 M reads) | WGS (30X) | 14 |
| DIPG4 | pDMG grade IV. 25% tumor cells. | WGS (90X) | RNASeq (100 M reads) | WGS (30X) | 13 |
| DIPG7 | pDMG grade IV, H3 K27M-mutated. 30% tumor cells. | WGS (90X) | RNASeq (100 M reads) | WGS (30X) | 10 |
| DIPG8 | pDMG grade IV, H3 K27M-mutated. 50% tumor cells. | WGS (90X) | RNASeq (100 M reads) | WGS (30X) | 10 |
| DIPG9 | pDMG grade IV, H3 K27M-mutated. >60% tumor cells. | WGS (90X) | RNASeq (100 M reads) | WGS (30X) | 10 |

**Table S4. Transcriptome-supported variants in pDMG sample**

| Sample ID | Gene | Variant_ID | TotCov_RNA | Cov_Alt_RNA | VarType | Variant_Score | HLA_binding_affinity |
| --- | --- | --- | --- | --- | --- | --- | --- |
| DIPG1 | OR52D1 | chr11:5489617:G:A | 0 | 0 | SNV | 31 |  |
|  | PIK3R1 | chr5:68293770:CTCAGTT:C | 366 | 161 | Deletion | 37,3 |  |
|  | SH3GL2 | chr9:17789536:C:G | 134 | 63 | SNV | 47,9 |  |
|  | ACVR1 | chr2:157770386:T:C | 154 | 74 | SNV | 68,2 | Yes |
|  | ADGRV1 | chr5:91150145:C:T | 237 | 118 | SNV | 41,4 | Yes |
|  | H3C2 | chr6:26031978:T:A | 0 | 0 | SNV | 22,1 | Yes |
|  | PLXNA3 | chrX:154466413:C:T | 45 | 0 | SNV | 21,6 | Yes |
| DIPG3 | KCNU1 | chr8:36923030:C:A | 3 | 0 | SNV | 25 |  |
|  | RP1 | chr8:54663832:C:A | 3 | 0 | SNV | 15,9 |  |
|  | COL4A5 | chrX:108665532:A:T | 35 | 17 | SNV | 51,9 |  |
|  | ZNF668 | chr16:31064161:C:T | 2 | 0 | SNV | 21,4 | Yes |
|  | H3-3A | chr1:226064434:A:T | 2748 | 1489 | SNV | 56,2 | Yes |
|  | OBSL1 | chr2:219556483:G:A | 80 | 13 | SNV | 24,5 | Yes |
|  | NIPBL | chr5:37022133:C:G | 138 | 53 | SNV | 40,9 | Yes |
|  | SENP6 | chr6:75711368:G:A | 428 | 169 | SNV | 42 | Yes |
|  | COL4A2 | chr13:110495342:G:T | 57 | 2 | SNV | 16,4 |  |
|  | SLC12A1 | chr15:48285123:G:C | 0 | 0 | SNV | 35,1 |  |
|  | MEF2A | chr15:99712661:G:A | 5 | 1 | SNV | 41,4 |  |
|  | NCOA6 | chr20:34758084:AGAG:A | 6 | 1 | Deletion | 16,7 |  |
|  | IL18RAP | chr2:102437251:G:A | 1 | 0 | SNV | 38,3 |  |
|  | CMTR1 | chr6:37474552:G:A | 25 | 7 | SNV | 18,5 |  |
| DIPG4 | RIMS2 | chr8:103912120:G:A | 1 | 1 | SNV | 44,7 |  |
|  | COL5A1 | chr9:134782697:G:T | 1 | 0 | SNV | 31,5 |  |
|  | RANBP6 | chr9:6015475:G:T | 10 | 3 | SNV | 31,4 |  |
|  | AL354733.3 | chr9:83975466:C:G | 903 | 407 | SNV | 68,6 |  |
|  | TP53 | chr17:7674221:G:A | 59 | 55 | SNV | 76,5 | Yes |
|  | PLXNB2 | chr22:50280523:C:T | 32 | 0 | SNV | 2 | Yes |
|  | CRIM1 | chr2:36356618:G:C | 5 | 1 | SNV | 37,3 | Yes |
|  | OR1B1 | chr9:122629424:G:T | 0 | 0 | SNV | 34,5 | Yes |
|  | NONE | chr12:19345927:A:C | 80 | 0 | SNV | 20,3 |  |
|  | MUC16 | chr19:8958564:T:C | 0 | 0 | SNV | 27,1 |  |
|  | NOL10 | chr2:10577680:C:T | 145 | 0 | SNV | 15,9 |  |
|  | CFP | chrX:47627171:G:A | 0 | 0 | SNV | 31,2 |  |
| DIPG7 | MYBPC3 | chr11:47339688:G:A | 0 | 0 | SNV | 33,7 | Yes |
|  | CENPT | chr16:67828335:G:A | 52 | 17 | SNV | 34,2 | Yes |
|  | TP53 | chr17:7675140:G:C | 21 | 16 | SNV | 56,6 | Yes |
|  | H3-3A | chr1:226064434:A:T | 1709 | 491 | SNV | 47,1 | Yes |
|  | PLXND1 | chr3:129605570:C:A | 0 | 0 | SNV | 16,5 | Yes |
|  | PFKFB1 | chrX:54951996:C:A | 2 | 0 | SNV | 21 | Yes |
|  | CD3G | chr11:118350614:G:A | 5 | 1 | SNV | 39 |  |
|  | TUFM | chr16:28844803:C:A | 598 | 46 | SNV | 18 |  |
|  | CHD9 | chr16:53242900:C:G | 12 | 8 | SNV | 49,3 |  |
|  | SLC4A3 | chr2:219631272:A:C | 11 | 0 | SNV | 35,8 |  |
| DIPG8 | POLE | chr12:132642348:C:T | 28 | 9 | SNV | 47,4 | Yes |
|  | OR4L1 | chr14:20060154:C:A | 0 | 0 | SNV | 34,1 | Yes |
|  | TP53 | chr17:7674953:T:C | 75 | 60 | SNV | 66,2 | Yes |
|  | H3-3A | chr1:226064434:A:T | 1717 | 743 | SNV | 57,1 | Yes |
|  | YIPF7 | chr4:44650022:C:T | 1 | 0 | SNV | 30,7 | Yes |
|  | COL5A1 | chr9:134727302:C:T | 3 | 2 | SNV | 49,2 | Yes |
|  | ERBB3 | chr12:56094403:G:A | 43 | 25 | SNV | 45,1 |  |
|  | SCN2A | chr2:165354456:G:A | 36 | 12 | SNV | 36,2 |  |
|  | PPP1R21 | chr2:48460099:A:G | 39 | 18 | SNV | 44,9 |  |
|  | PPP2R2B | chr5:146878069:C:T | 171 | 68 | SNV | 47 |  |
|  | CSPP1 | chr8:67115971:C:A | 51 | 24 | SNV | 38,5 |  |
| DIPG9 | AC012676.1 | chr16:4337131:TG:T | 3 | 0 | Deletion | 22,8 | Yes |
|  | CAMTA2 | chr17:4970392:G:T | 7 | 6 | SNV | 44,5 | Yes |
|  | H3-3A | chr1:226064434:A:T | 1010 | 370 | SNV | 41,6 | Yes |
|  | ASXL1 | chr20:32434638:AG:A | 8 | 4 | Deletion | 35,5 | Yes |
|  | SLC35G2 | chr3:136854501:G:A | 56 | 22 | SNV | 44,7 | Yes |
|  | TRAM1L1 | chr4:117084477:G:T | 102 | 46 | SNV | 48,5 | Yes |
|  | NOBOX | chr7:144397490:G:A | 1 | 0 | SNV | 26 | Yes |

**Table S5. HLA class I typing of pDMG samples and GBMC6**

| Sample ID | A1 | A2 | B1 | B2 | C1 | C2 |
| --- | --- | --- | --- | --- | --- | --- |
| DIPG1 | A*02:01 | A*02:01 | B*07:02 | B*07:02 | C*07:02 | C*07:02 |
| DIPG3 | A*24:02 | A*24:02 | B*27:05 | B*40:01 | C*03:04 | C*07:02 |
| DIPG4 | A*02:01 | A*26:08 | B*44:02 | B*44:02 | C*05:01 | C*05:01 |
| DIPG7 | A*01:01 | A*25:01 | B*18:01 | B*38:01 | C*12:03 | C*12:03 |
| DIPG8 | A*01:01 | A*03:01 | B*35:01 | B*51:01 | C*04:01 | C*15:02 |
| DIPG9 | A*02:01 | A*03:01 | B*44:02 | B*51:01 | C*07:02 | C*05:01 |
| GBMC6 | A*02:01 | A*03:02 | B*13:02 | B*35:01 | C*03:03 | C*06:02 |

**Table S6: Comparison of neoantigens candidates identified by PIOR score pipeline and HLA binding prediction across pDMG Samples**

| Sample ID | PIOR score | HLA binding affinity |
| --- | --- | --- |
| DIPG1 | chr2:157770386:T:C | chr2:157770386:T:C |
|  | chr9:17789536:C:G |  |
|  | chr5:91150145:C:T | chr5:91150145:C:T |
|  | chr5: 68293770:CTCAGTT:C |  |
|  | chr11:5489617:G:A |  |
|  | chr6:26031978:T:A | chr6:26031978:T:A |
|  | chrX:154466413:C:T | chrX:154466413:C:T |
| DIPG3 | chr1:226064434:A:T | chr1:226064434:A:T |
|  | chrX:108665532:A:T |  |
|  | chr17:7675097:A:ACCT |  |
|  | chr3:179218303:G:A |  |
|  | chr18:32770315:A:C |  |
|  | chr6:75711368:G:A | chr6:75711368:G:A |
|  | chr5:37022133:C:G | chr5:37022133:C:G |
|  | chr12:43434263:T:G |  |
|  | chr8:36923030:C:A |  |
|  | chr2:219556483:G:A | chr2:219556483:G:A |
|  | chr16:31064161:C:T | chr16:31064161:C:T |
|  | chr16:31380040:C:T |  |
|  | chr4:68827542:A:G |  |
|  | chr8:54663832:C:A |  |
| DIPG4 | chr17:7674221:G:A | chr17:7674221:G:A |
|  | chr9:83975466:C:G |  |
|  | chr8:103912120:G:A |  |
|  | chr15:99712661:G:A |  |
|  | chr2:102437251:G:A |  |
|  | chr2:36356618:G:C | chr2:36356618:G:C |
|  | chr15:48285123:G:C |  |
|  | chr9:122629424:G:T | chr9:122629424:G:T |
|  | chr9:134782697:G:T |  |
|  | chr9:6015475:G:T |  |
|  | chr6:37474552:G:A |  |
|  | chr20:34758084:AGAG:A |  |
|  | chr13:110495342:G:T |  |
|  |  | chr22:50280523:C:T |
| DIPG7 | chr17:7675140:G:C | chr17:7675140:G:C |
|  | chr1:226064434:A:T | chr1:226064434:A:T |
|  | chr16:67828335:G:A | chr16:67828335:G:A |
|  | chr11:47339688:G:A | chr11:47339688:G:A |
|  | chrx:47627171:G:A |  |
|  | chr19:8958564:T:C |  |
|  | chrX:54951996:C:A | chrx:54951996:C:A |
|  | chr12:19345927:A:C |  |
|  | chr3:129605570:C:A | chr3:129605570:C:A |
|  | chr2:10577680:C:T |  |
| DIPG8 | chr17:7674953:T:C | chr17:7674953:T:C |
|  | chr1:226064434:A:T | chr1:226064434:A:T |
|  | chr16:53242900:C:G |  |
|  | chr9:134727302:C:T | chr9:134727302:C:T |
|  | chr12:132642348:C:T | chr12:132642348:C:T |
|  | chr11:118350614:G:A |  |
|  | chr2:219631272:A:C |  |
|  | chr14:20060154:C:A | chr14:20060154:C:A |
|  | chr4:44650022:C:T | chr4:44650022:C:T |
|  | chr16:28844803:C:A |  |
| DIPG9 | chr4:117084477:G:T | chr4:117084477:G:T |
|  | chr5:146878069:C:T |  |
|  | chr12:56094403:G:A |  |
|  | chr2:48460099:A:G |  |
|  | chr3:136854501:G:A | chr3:136854501:G:A |
|  | chr17:4970392:G:T | chr17:4970392:G:T |
|  | chr1:226064434:A:T |  |
|  | chr8:67115971:C:A |  |
|  | chr2:165354456:G:A |  |
|  | chr7:144397490:G:A | chr7:144397490:G:A |
|  |  | chr1:226064434:A:T |
|  |  | chr20:32434638:AG:A |
|  |  | chr16:4337131:TG:T |

**SUPPLEMENTAL FIGURE LEGENDS**

**Supplemental Figure 1: Heatmap of predicted drug sensitivities across the pDMG cohort.** Heatmap visualizing the computed Drug Sensitivity Score (DSS) Z-scores for multiple pharmacological agents (e.g., Camptothecin, Dinaciclib, Panobinostat) across the evaluated DIPG samples and corresponding normal reference tissues from public databases (OpenPBTA, GTEx).

**Supplemental Figure 2: Predicted drug sensitivity by broad cancer group.** Boxplots demonstrating the distribution of Drug Sensitivity Scores (DSS) for candidate therapeutics (e.g., Dactinomycin, Docetaxel, Thapsigargin) across broadly defined reference cancer groups, including DIPG, Medulloblastoma, Ependymoma, and others.

S**upplemental Figure 3: Predicted drug sensitivity mapped to molecular subtypes.** Boxplots detailing the predicted Drug Sensitivity Scores (DSS) for candidate agents relative to specific molecular subtypes (e.g., DMG, H3 K28; HGG, H3 wildtype; EPN, PF A).

**Supplemental Figure 4: Predicted drug sensitivity by specific cancer type.** Boxplots detailing the predicted Drug Sensitivity Scores (DSS) categorized by specific cancer type classifications, evaluating the variance in predicted response for agents such as Bortezomib, Erlotinib, and Paclitaxel.

**Supplementary Figure 5: HLA expression references.** (A) Tissue-specific expression profiles of HLA-A, HLA-B, and HLA-C across different human tissues, measured in RPM. (B) Representative flow cytometry histograms displaying surface HLA-A2 expression levels across samples. Overlay histograms of HLA-A2 staining for GBMC6 (black), Huh7 A2– (red), Huh7 A2+ (pink), and primary dendritic cells (blue).
